# Three Years with the Army of the Potomac—A Personal Military History

**Published:** 1911-12

**Authors:** William Warren Potter

**Affiliations:** Buffalo, N. Y., Brevet Lieutenant Colonel, U. S. Volunteers; Surgeon in Charge of First Division Field Hospital Second Army Corps; Surgeon 57th Regiment New York Volunteers; Assistant Surgeon 49th Regiment New York Volunteers; Recorder Second Division Hospital Sixth Army Corps, etc., etc.


### Three Years with the Army of the Potomac—A Personal Military History

By William Warren Potter, M.D., Buffalo, N. Y., Brevet Lieutenant Colonel, U. S. Volunteers; Surgeon in Charge of First Division Field Hospital Second Army Corps; Surgeon 57th Regiment New York Volunteers; Assistant Surgeon 49th Regiment New York Volunteers; Recorder Second Division Hospital Sixth Army Corps, etc., etc.

### ON THE "SACRED SOIL" AGAIN.

We have heard distant firing in Virginia at intervals all day, which appears to be in the vicinity of Snicker's Gap, and have been under orders to move since morning, but night finds us still here. We have, however, received final orders to march at six A. M. tomorrow. Tom Dewey did not go on recruiting service as expected, but sent in his resignation a few days ago. I believe he expects to enter the artillery service. I am sorry to have him leave the 49th, for he is one of our best officers. Adjutant Selkirk departs for Buffalo in the morning, on recruiting service in Dewey's stead.

November 3. We crossed the Potomac on a pontoon bridge at Berlin this morning, and have marched about seventeen miles today, reaching Purcellville where we have encamped for the night. I was chilled through on the march, as the weather was very cold; our stove comes in place most delightfully tonight. Tomorrow will be Election Day in New York, and I regret that I cannot be present at the polls to cast my vote against Seymour, who is the Democratic candidate against General Wadsworth. On the 4th we reached Union, Virginia, (What's in a name?) and went into camp at three o'clock P. M. for the night, as we supposed, only; but we remained there until one P. M. of the 5th, when we moved five miles farther, and encamped in the woods after dark, where we were without tents, and a colder night we have not seen.

November 6. We marched twelve miles which brought us to White Plains, on the Manassas Gap R. R. I lost my gloves, and so had cold hands today. General Seth Williams told Dr. Mulford yesterday, so the latter informs me, that the Sixth Corps would be given the reserve position, and would always be near McClellan's headquarters. Dr. Mulford also says he intends to resign from the 33d, and enter the Volunteer Staff Corps. I shall be sorry to have him leave our Brigade; but so it ever is, our friends are constantly looking to advancement, here as well as in civil life. The changes in the personnel of the Army, through promotion, casualties, and other causes, is something remarkable. One scarcely knows his friends from campaign to campaign. Mart Clark of the 21st came on the 7th, and spent the night in our camp.

November 8. It is cold and snowed all day yesterday, but our stove makes it comfortable for ourselves, as well as others who call. We were thronged in our quarters yesterday, and Colonel Bidwell spent the entire day with us. On Sunday, the 9th, we moved from White Plains to New Baltimore, a distance of eight miles, over a mountainous country and in a searching wind. General McClellan has been removed from command, and General Burnside appointed thereto.

EXIT MC CLELLAN—ENTER BURNSIDE.

November 11. Today, Tuesday, General McClellan took leave of the Army, and the Sixth Corps was paraded to bid him farewell. He bade his adieus to the music of drums, cannon, and cheers. He looked sad as he rode along the lines, and many were moved to tears as he passed. Altogether it was sad and affecting. The news of Seymour's election, too, coming at this time, is especially dispiriting. We are still (13) at New Baltimore, and I presume our quietude is to enable the new Commander to seize the reins with a firm hold. We have heard firing beyond Warrenton today, but know nothing of its import.

Novemebr 15. Still quiet at New Baltimore, and the weather is delightful. It is rumored that Franklin is to have command of a Grand Division, to consist of two or three corps, in which case Smith will have the Sixth Corps, and Howe the Second Division. Captain Wheeler is reported at Warrenton, and if so he will soon be here. He has been absent since we were at Fort Monroe, and his long absence has furnished occasion for unfavorable comment in certain quarters.

### THE FREDERICKSBURG CAMPAIGN.

November 17. We left New Baltimore early yesterday morning, and encamped last night about two miles east of Catlett's Station. This morning we started again at 6.30 o'clock, and marched about fifteen miles; which brings us to a point about fifteen miles N. N. W. of Fredericksburg. On the 18th we moved to Stafford C.H., and camped in a dry woods near an old brick church. Our base of supplies is at Acquia Creek, seven miles distant. Captain Wheeler has returned to the regiment, and on the 22d had a trial for absence without leave before a military commission. I was, of course, summoned as a witness. The weather is bad and we are "Stuck in the mud."

November 24. The Grand Division organizations have been perfected and Franklin has the Left Grand Division, Smith the Sixth Corps, and Howe the 2d Division. The Left Grand Division comprises the 1st and 6th Corps, the 1st being commanded by Reynolds. The 21st is in the 1st Corps, and I paid that regiment a visit yesterday in company with Dr. Mulford and Captain Wheeler, where I saw Mart Clark, but spent most of my time with Captain Al. Wheeler, who is about the only officer left there of my particular acquaintance. Captain W. F. Wheeler has been dismissed from the service for absence without leave. He was one of our best officers, and his dismissal is one of those unfortunate combinations of circumstances that often happen in the service. I hope the order will be revoked, as it is, in my opinion, a most unjust and cruel judgment.. I believe that there was some neglect to present the case properly before the Commission, and that, further, a re-hearing should be demanded by Captain Wheeler on that ground.

November 26. Lieutenant Bliss returned from leave, and brought me a package from home containing gloves, stockings, etc. Tomorrow will be Thanksgiving Day, and we are making some extra preparations for dinner on that account, though nothing very elaborate can be served, as we are not in a region of abundance. However, we shall have a roast turkey and some potatoes, both sweet and Irish, so I fancy we'll get along without starving some way. The weather is unfavorable for military operations, the frequent rains having made the roads almost impassable.

November 30. This being the last day of the month I am busy with the monthly reports, which I have always to make up. Dr. Hall is now Surgeon-in-Chief of the Brigade, Dr. Mulford, who was holding the place by virtue of seniority, having resigned and gone North. On Friday, the 28th, I went to Acquia Creek Landing to draw medical supplies for the Brigade, and saw Tom Dewey off for home, his resignation having been accepted at last. He was one of our best subaltern officers and Colonel Bidwell told me he would recommend him for Captain of Company D, if he would remain. I hear that Captain McLean is on recruiting service in Elmira. Lieutenant Charles O. Shepard, of the 82d New York Volunteers, has also resigned, and he went North on the same boat with Tom Dewey; so I bade them good-bye at the wharf, wishing them much joy upon their release from the dangerous and irksome duties of field service. Dr. Oliver Adams, brother to Dr. N. H., came down to the Army with Lieutenant Bliss the other day, and I understand he has been appointed Assistant Surgeon in one of the New York Regiments, the 78th I think. The weather is better than at the last record, but the roads are still horrible, impassable for heavy trains and artillery. Colonel Bidwell's health is quite poor, and I fear will break down completely if we have an active campaign.

December 4. The weather is bright, and we are under orders to move at eleven o'clock A. M. today, rumor says to Belle Plain, below Acquia Creek Landing. December 6: we left our camp near Stafford C. H. day before yesterday as expected, and as our baggage train did not arrive until late into the night, we were without tents. Yesterday we moved four miles again, but the artillery and trains did not get up until noon of the 7th. I was out of doors all night the 5th, without cover of any kind excepting my overcoat, and it was the most severe night I have experienced in the service. I revolved before a big camp fire all night long, but it was so cold I could not sleep a wink. The ground is covered with snow, and the ambulances are stuck in the mud for miles along the road. My horses have had no grain for two days, and I have lived during that time on hard bread, with short allowance at that. Four men died in the ambulances yesterday while we were on the march. Just before our last move I was detailed to the medical charge of the Division ambulance train when on a march, which adds to my duties and responsibilities very much. It is the duty of the Medical Officer in charge of the train to examine all passes given to the sick, and to pick them up if he approves of their authority to "Fall out," and to minister to their wants or necessities during such march.

December 8. We are at Belle Plain, or near there, but quiet—in fact, we cannot move on account of the mud. This is the most trying campaign we have experienced, yet I am in good health and gaining in flesh all the time. December 10. Still at the same place, though we have been under orders to march at a moment's notice since yesterday, with three days' cooked rations and sixty rounds of ammunition, which looks like a fight. Lieutenant Gilman has gone home on leave; he is a faithful, deserving officer, and I am glad he has been allowed this indulgence.

December 13. I have been appointed Recorder of the 2d Division Hospital, Sixth Corps. We moved down to the Rappahannock night before last, and crossed the river yesterday morning early on two pontoon bridges, about two miles below the town of Fredericksburg. Our hospital is located at Mansfield, Bernard's Mansion, on the South Bank of the river near the bridge head. We had a battle today in which the 49th lost ten or twelve men, mostly wounded. Lieutenant Bliss was slightly wounded in the head—the only officer struck. Sumner's right Grand Division suffered a heavy loss, we hear, having made repeated assaults upon the enemy's strong position in front of the town. It is expected the battle will be renewed in the morning. I sent my letters by the correspondents of the *N. Y. Times* and *Tribune,* who come to the hospital to get the lists of killed or wounded, and accord me that privilege in return for courtesies shown them. This is a great advantage, as we have no mail facilities during a battle.

December 14. The battle was not renewed today as expected, General Burnside having countermanded the order which he first gave, to do so. General George D. Bayord, of the Cavalry, was struck by a solid shot while in conversation with General Franklin, and I had him brought in immediately. He was on the lawn in front of the hospital when it happened, and I was standing on the steps at the time. The shot rolled along and expended its force against the foundation walls of the building. The injury is a mortal one, and it is doubtful if he lasts out the day.

December 16. We remained quiet during the 14 and 15, except the usual skirmish firing and an occasional gun, and last night recrossed the Rappahannock in safety. We are now on the north bank about one and a quarter miles from the river, having been busy all night in removing and caring for the wounded. We seem to have been fortunate in extracting ourselves from a bad box. The battle of Fredericksburg will always stand in history, as a monument to the folly, and incompetency, of the second commander of the Army of the Potomac.

December 18. I was ordered to take the wounded of the 2d Division to Washington. They were collected at Falmouth and put aboard of the cars, whence they were transported by rail to Acquia Creek, where we arrived about eight o'clock in the evening. Two transports at anchor off the landing were brought to the wharf, and the transfer of the wounded to the boats was accomplished by three o'clock A. M., whereupon we steamed for Washington, arriving there by seven o'clock. The wounded were fed by the Sanitary Commission's agents while they were transferring to the ambulance, but it was three o'clock P. M. before they were all off for their various hospitals. Up to this time I had not eaten anything since leaving Falmouth the night before, so I was nearly famished; but I made haste for a good hotel where I soon obtained the needed food and rest. The troops had not been paid since June, and I was quite out of money; so I availed myself of this opportunity to visit the Pay Department, where I drew four months' pay on the 20th, sending $300.00 home by the first express.

### FAREWELL TO THE 49TH.

I remained in Washington until the 23d, for rest and recuperation, starting back for the Army on the latter date. Arriving at Falmouth late in the evening, I rode to Franklin and Smith's headquarters in one of General Smith's headquarter wagons which I found at the station. Here I obtained supper about nine o'clock, and remained over night as the guest of Dr. Basil Norris, the Medical Director of the Left Grand Division. Next morning, the 24th, I made my way on foot to the camp of the 49th, about a mile and a half distant, and on my arrival there I found a letter from Dr. Vanderpoel, Surgeon General of the State of New York, promoting me to be Surgeon of the 57th Regiment New York Volunteers.

Thus it came about that, after fifteen months' service as Assistant Surgeon, I found myself suddenly invested with the full rank of Surgeon, and not yet quite twenty-four years old. This seemed to present the opportunity for me to obtain a leave of absence, which, as I had not been thus indulged since entering the service, I thought myself entitled to. Therefore, armed with my letter of promotion, I took myself back to Grand Division Headquarters, and presented the case to Dr. Norris, knowing full well he would do all in his power to aid me. The orders at this time in force on the subject of leaves of absence for officers were very strict, but Dr. Norris took the letter in to General Franklin and stated the case to him. Franklin, though a disciplinarian, was a kind officer, but was powerless to act, no matter how deserving the case, in the face of positvie orders; and this was the report Dr. Norris brought back to me. I then suggested to Dr. Norris that I could resign as Assistant Surgeon, to accept promotion. He asked Franklin how that would do. The General readily assented to such a plan, stating he would approve such a paper, and that I might take the responsibility of going home upon that authority, though suggesting that the visit be a short one.

These negotiations consumed the day the 24th, and as the next day was Christmas nothing was done; but on the 26th I sent up my resignation as indicated, which came back duly approved on the 27th, and the necessary order was issued. (This paper will be found among my military files.) In the meantime, on Christmas Day, I visited the 136th New York, which was lying near Falmouth, seven miles away, where I met many old acquaintances—Gad Parker, John McCray, John Boyd, Dr. Edwin Amsden, and others. Dr. A. was anxious to be promoted to Surgeon, as he had been in service sometime; and I am persuaded that he did not altogether enjoy himself with his superior, who was Dr. B. H. Hovey, now of Rochester. I engaged to speak a word for him to Surgeon General Vander Poel, in case I got away as expected. Another object of this visit to Falmouth, was to ascertain something of the 57th, which I understood was encamped near there. I saw the regiment in its camp, and learned that it belonged to the First Division (Hancock's) Second Corps. I did not pay it a call, lest, if I did, my plans for going home would be spoiled.

Sunday was the 28th, and I spent the day in packing up, bidding good-bye to the officers and men, and in otherwise preparing to leave Monday morning, December 29th, bright and early, I bade adieu to the 49th after fifteen months' continuous service with it, and sped my way to Falmouth Station to take the train to Acquia Creek. When the time finally came to depart I was loth to go, for I had many friends who were tried and true, whereas, in my new field, I was a total stranger, not having a solitary acquaintance in the regiment. However, go I must, no matter how regretfully, and go I did, with many a kind expression from both officers and men of the old 49th. From Acquia the boat carried me up the Potomac to Washington, where I arrived near three o'clock P. M. I was anxious to reach the Pay Department before the office should close, so took a carriegs at the Sixth Street wharf and drove rapidly to the office, where I had my resignation paper properly endorsed for pay by E. H. Brooke, the Chief Clerk. He referred me to Major Rochester (now Paymaster General) for payment; but by the time I reached his office it was too late. He was just closing up for the day and asked me to call the next morning, when he would pay me my balance for November and December, 1862. But I was impatient, and took the evening train for New York without waiting for pay. I reached New York on the morning of December 30, spent the day there in obtaining a new uniform to correspond with my increased rank, Major of Cavalry, and proceeded to Albany the same evening, where I arrived at ten o'clock P. M. I took rooms at the Delavan House, and next morning proceeded to the Capitol to interview the Surgeon General, Dr. S. Oakley Vander Poel.

I found Dr. Vander Poel in his office at ten o'clock A. M., and had a pleasant visit with him for half an hour. This was the last day of the year, and also his last day in office, as Governor-Elect Seymour would take office the next day, and, of course, appoint a Democrat to succeed Vander Poel. I took occasion to thank him for his kindness to me in various ways, and particularly in looking after my interests in the way of promotion before he left office. He replied that it was his policy, as far as possible, to promote all the old Assistant Surgeons before retirement, and that my record was such as to merit what I had received. I then told him how I had succeeded in getting away from the Army for a visit home; whereupon, he smilingly said that I had better make the visit as brief as possible, so as to give his successor no cause for complaint. I said I would return at the end of twenty days, which was the usual length of leaves of absence to officers. Finally, I asked him to promote Dr. Amsden before retiring, but he said that would now be impossible, as it might look like forestalling the action of the new Surgeon General if he made appointments of promotions on the last day of office, whereupon the interview terminated. I enjoyed Dr. Vander Poel's personal friendship in after years, meeting him often at the State Medical Society, and we rode together from Albany to New York only a few days before his death, which occurred March 13, 1886. I took the afternoon train for the West, arriving at Lancaster about midnight, where I persuaded the conductor to let me off, as the train did not make a regular stop there. It was nearly one o'clock when I reached the house and awoke my wife who, up to this time was unaware either of my promotion or of my coming. My son Frank was then nearly three years old, and even at that late hour was aroused by my arrival, when we all had a happy meeting.

